# Developing immunotherapies through glycosylation reprogramming to combat solid cancers

**DOI:** 10.3389/fimmu.2026.1824624

**Published:** 2026-08-06

**Authors:** Toshihiko Toyofuku, Atsushi Kumanogoh

**Affiliations:** 1 Department of Immunology and Molecular Medicine, Graduate School of Medicine, The Center of Medical Innovation and Translational Research, Osaka University, Suita, Osaka, Japan; 2 Department of Respiratory Medicine and Clinical Immunology, Graduate School of Medicine, Osaka University, Osaka, Japan; 3 Laboratory of Immunopathology, WPI Immunology Frontier Research Center, Osaka University, Suita, Osaka, Japan; 4 Integrated Frontier Research for Medical Science Division, Institute for Open and Transdisciplinary Research Initiatives, Osaka University, Suita, Osaka, Japan

**Keywords:** cancer, CAR-T cells, immune checkpoint inhibitor, immunotherapy, metabolism

## Abstract

Cancer immunotherapies, including immune checkpoint inhibitors and adoptive cell transfer, have shown great efficacy and promise against hematological malignancies. However, the value of these therapies remains inconclusive in the context of solid cancers and is limited by several obstacles, including difficulty in identifying cancer-specific antigens, limited immune cell trafficking and infiltration into tumors, and the immunosuppressive effects of the tumor microenvironment. To further optimize the clinical effects of immunotherapies, many studies have focused on modifying metabolic programs, especially glycosylation, in cells. Glycosylation, which adds a diversity of carbohydrate structures (glycans) to surface molecules on virtually all cells, regulates stimulatory and inhibitory signaling pathways. Therefore, modification of key glycosylation pathways represents a logical approach for the development of new therapeutic strategies for treating solid cancers. We review how specific glycans regulate the anticancer activities of immune cells and immunosuppressive effects of cancer cells in cancer immunotherapy. In addition, we highlight T cells with NK cell properties *ex vivo* primed by N-glycosylation inhibitor and their advantageous functions for treatments of solid cancers.

## Introduction

Cancers evade the immune system in many ways in order to survive in the host. The immune system’s role in identifying and eliminating tumor cells has paved way for immunotherapy, a strategy designed to enhance anti-tumor immunity. One such mechanism is the promotion of T cell exhaustion, marked by the progressive acquisition of inhibitory receptors (1–3). To overcome T cell exhaustion, blockade of inhibitory receptors by antibodies, so-called immune checkpoint inhibitor therapy, successfully enhances T cell activity against cancer. However, clinical trials have failed to demonstrate the efficacy of this therapy for patients with solid cancers. A strategy designed to enhance tumor antigen-specific immunity rather than non-specific enhancement via immune checkpoint inhibitor therapy has gained more interest. An alternative therapeutic approach targeting tumor-specific antigens that can prevent drug resistance and cancer relapse is the adoptive T cell transfer into patients. Anticancer T cell immunotherapy consists of engineering immune cells to express cell surface receptors such as Chimeric Antigen Receptor (CAR) against tumor-specific antigens. The transfer of engineered T cells expressing CAR into patients, so-called CAR-T cell therapy is capable of recognizing antigens expressed on the surface of tumor cells and destroying them (4). CAR-T cell approaches have demonstrated great potential in mediating long-lasting responses against hematologic cancers such as lymphoblastic leukemia (5–7). CAR-T cells have also been used to treat solid tumors; however, curative effects are lacking compared with those observed in lymphoblastic leukemia (8, 9). Several major limitations have been proposed, including the difficulty in identifying tumor-specific antigens suitable for immunotherapy, difficulty in tumor infiltration by the cytotoxic T cells, and insufficient survival of immune cells in the immunosuppressive tumor microenvironment (10). Hence, there is an urgent need to further optimize CAR-T therapy to resolve existing issues with solid tumors. One approach is optimizing CAR constructs, which effectively combines the targeting precision of an antibody with the cytotoxic capabilities of a T cell. Another approach is the novel *ex vivo* manufacturing of CAR-T cells or alternative cytotoxic immune cells for CAR-T cell therapy (11). Tumor cells are characteristically different from their healthy counterparts. Factors such as hypoxia, oncogenic mutations, and altered signaling induce up-regulation of anabolic processes and suppression of catabolic pathways in tumor cells. While each type of T cells exhibits a characteristic metabolic phenotype, cytotoxic T cells up-regulate catabolic pathways; conversely, naïve and memory T cells up-regulate anabolic pathways. The *ex vivo* manufacturing of T cells should maximize a high percentage of memory T cells, ultimately resulting in superior longevity and anti- cancer potential for CAR-T immunotherapy. Furthermore, there should be interaction between cytotoxic T cells and tumor cells established through cell surface receptors, which are modified with various glycosylation patterns. The increasing understanding of the cell-specific glycosylation patterns has expanded research focus to modification of glycans on CAR-T cells to overcome the obstacles in immunotherapy for solid tumors.

Herein, we summarize the glycolysis and glycosylation pathways operating in immune cells and cancer cells and review the current status and future prospects of immunotherapies, including immune checkpoint inhibitor therapy and CAR-T cell therapy, as well as the focus on how glycosylation based modifications may enhance their efficacy. We also discuss the possibility that modification of N-glycans could differentiate T cells into more cytotoxic NK-like T cells for the promotion of cell-based cancer immunotherapy.

## Glycolysis and glycosylation of immune cells and tumor

### Glycolytic pathway in immune cells

**Figure 1:**
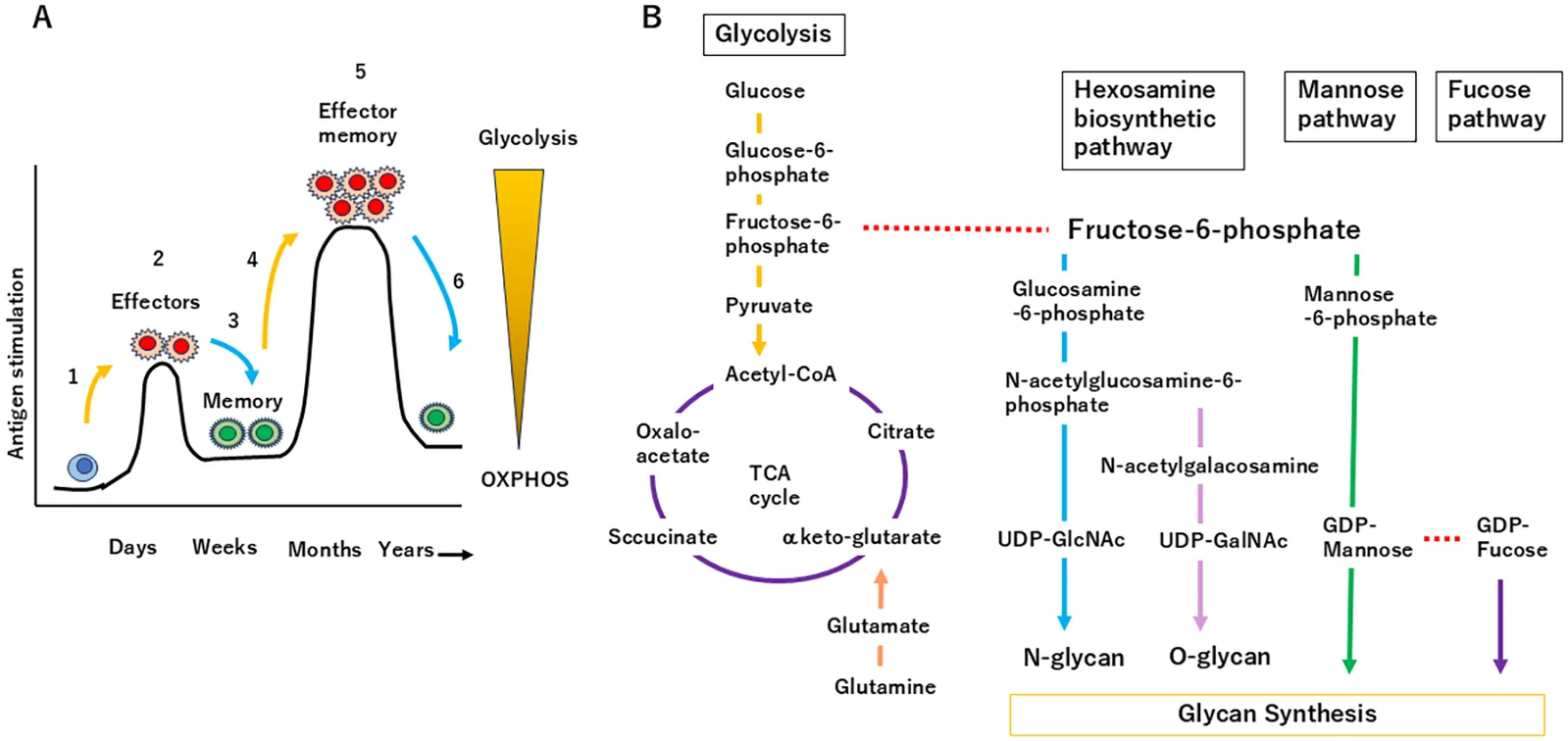
Dependence of T cell differentiation on distinct metabolic states and the overview of major glucose-utilizing pathways. **(A)** Descriptions of T cells based on their differentiation and memory status (1) Naïve T cells breakdown glucose and efficiently break it down through the tricarboxylic acid (TCA) cycle and oxidative phosphorylation (OXPHOS) to survive. (2) Upon primary exposure to an antigen, naive T cells differentiate into effector T cells; they shift towards the use of amino acids and glucose. (3) After clearing antigens, many effector T cells die. A fraction of surviving T cells forms memory T cells, which adapt towards OXPHOS. (4) These memory T cells survive for months to years until they encounter a similar antigen. (5) Upon re-encounter of the same antigen, they rapidly become effector T cells and more efficiently engage in glycolysis and amino acids usage to robustly proliferate and secrete cytokines. (6) The cells that survive maintain usage of OXPHOS to persist long-term. **(B)** Diagram of four major glucose-utilizing pathways; the glycolysis and hexosamine biosynthetic, and fucose pathways. All pathways share the first steps converting glucose to glucose-6-phosphate and subsequent conversion to fructose-6-phosphate. Glutamine: fructose-6-phosphate amidotransferase (GFAT) converting fructose-6-phosphate to glucosamine-6-phosphate catalyzes the rate-limiting step of the hexosamine biosynthetic pathway. UDP-GlcNAc is a vital substrate for N-glycan synthesis. UDP-GalNAc is a vital substrate for O-glycan synthesis. Mannose-6-phosphate is generated from glucose or mannose imported from the extracellular space. Finally, GDP-Mannose coupled to the guanosine diphosphate (GDP), is used as a substrate for N-glycan synthesis or substrate for synthesizing GDP-fucose, another prevalent monosaccharide in N-glycan.

T cells use distinct metabolic pathways based on their differentiation and memory status (12–14) (**Figures 1A, B**). Glucose is metabolized into pyruvate through an anaerobic glycolysis pathway, after which pyruvate enters the mitochondria and is converted to acyl CoA, which generates ATP via TCA cycle and oxidative phosphorylation (OXPHOS). Naïve T cells can persist throughout the host’s lifetime. Naïve T cells, dependent on IL7 which promotes glucose uptake into cells, rely on OXPHOS to survive in their resting state. Upon primary antigen exposure, naïve T cells become activated upon T cell receptor (TCR) engagement with antigens presented by the major histocompatibility complex (MHC) on antigen-presenting cells with costimulatory signals. Instead of engaging in the highly energetically favorable OXPHOS, effector T cells use glycolytic pathway with minimal engagement of mitochondrial respiration to effectively secrete cytokines, such as interferon-γ (IFN-γ) and tumor necrosis factor-α (TNF-α). After antigen clearance, most effector T cells undergo apoptosis; however, a subset survives and differentiates into memory T cells, which provide long-term protection against reinfection or tumor relapse. Compared with effector T cells, memory T cells exhibit enhanced mitochondrial biogenesis, increased mitochondrial fusion, and greater reliance on OXPHOS. Upon re-encounter with the same antigen, memory T cells rapidly reacquire effector functions to clear the insults. Effector T cells derived from memory produce cytolytic cytokines more efficiently than those derived from naïve T cells, partly through improved coupling between glycolytic enzymes and mitochondrial machinery, allowing rapid glucose utilization during secondary antigen exposure. Most effector memory T cells undergo apoptosis, but a fraction persists through OXPHOS-dependent metabolism. Thus, T cell development relies on the metabolites in glycolysis pathways. Glutamine is a substrate for the TCA cycle, and its metabolism is augmented once naïve T cells are activated (15). Glutamine is required for effector T cell differentiation and function, and its depletion increases the percentage of central memory T cells (15). Hence, the core mechanism that induces increase in OXPHOS and appropriate inhibition of glycolysis *in vitro* can lead to a high percentage of stem cell memory T cells and central memory T cells, ultimately resulting in superior longevity and anti- cancer potential for CAR-T immunotherapy.

### Glycolytic pathway in tumor cells

In contrast to normal cells, many tumor cells exhibit enhanced glycolysis and relatively reduced OXPHOS capacity (16). The glycolytic contribution to total ATP production does not exceed 50–60%, and OXPHOS still substantially contributes to ATP production in tumor cells. Under hypoxic conditions, however, the contribution of OXPHOS may decline to approximately 30%. Hypoxia is a frequent feature of solid tumors, and enhanced glycolysis provides a growth advantage in such environments. Glycolysis produces lactate, which is exported into the extracellular space, leading to acidification of the tumor microenvironment. This acidic milieu promotes cancer cell invasion and metastasis (17). In certain tumor cells, glutaminolysis serves as an alternative pathway for energy production. Glutamine is converted into a-ketoglutarate, which enters the TCA cycle to replenish intermediates and energy for cell growth. Oncogenic alterations further drive metabolic reprogramming in cancer. Mutations in oncogenes such as Ras and Myc, as well as loss or mutation of tumor suppressor p53, play a central role in aerobic glycolysis (18, 19). Hypoxia-inducible factor (HIF), a transcription factor induced under low-oxygen conditions, plays critical roles in shifting from OXPHOS to the glycolytic phenotype in tumor cells (20). Oncogene Ras mutations stimulate PI3K/Akt/mTOR signaling pathway, enhancing tumor cell proliferation and inducing HIF expression to support survival under hypoxic stress. Oncogene Myc, frequently overexpressed in human cancers, functions as a master transcription factor which regulates over 15% of human genes, including cell cycle, metabolism, ribosome biogenesis, RNA, mitochondrial biogenesis, and apoptosis. Myc stimulates aerobic glycolysis by upregulating glucose transporter and enhancing glutamine metabolism. Tumor suppressor p53, a transcription factor, inhibits glycolysis and promotes OXPHOS and blocks the accumulation of HIF in normoxia and hypoxia. However, mutant p53, one of the most common mutations of genes in human cancers, induces aerobic glycolysis and HIF accumulation. Thus, mutant p53 combined with HIF1 and c-Myc has been described as the triad of transcription factors responsible for the glycolytic phenotype in cancer (18).

### Glycosylation pathway in cells

**Figure 2:**
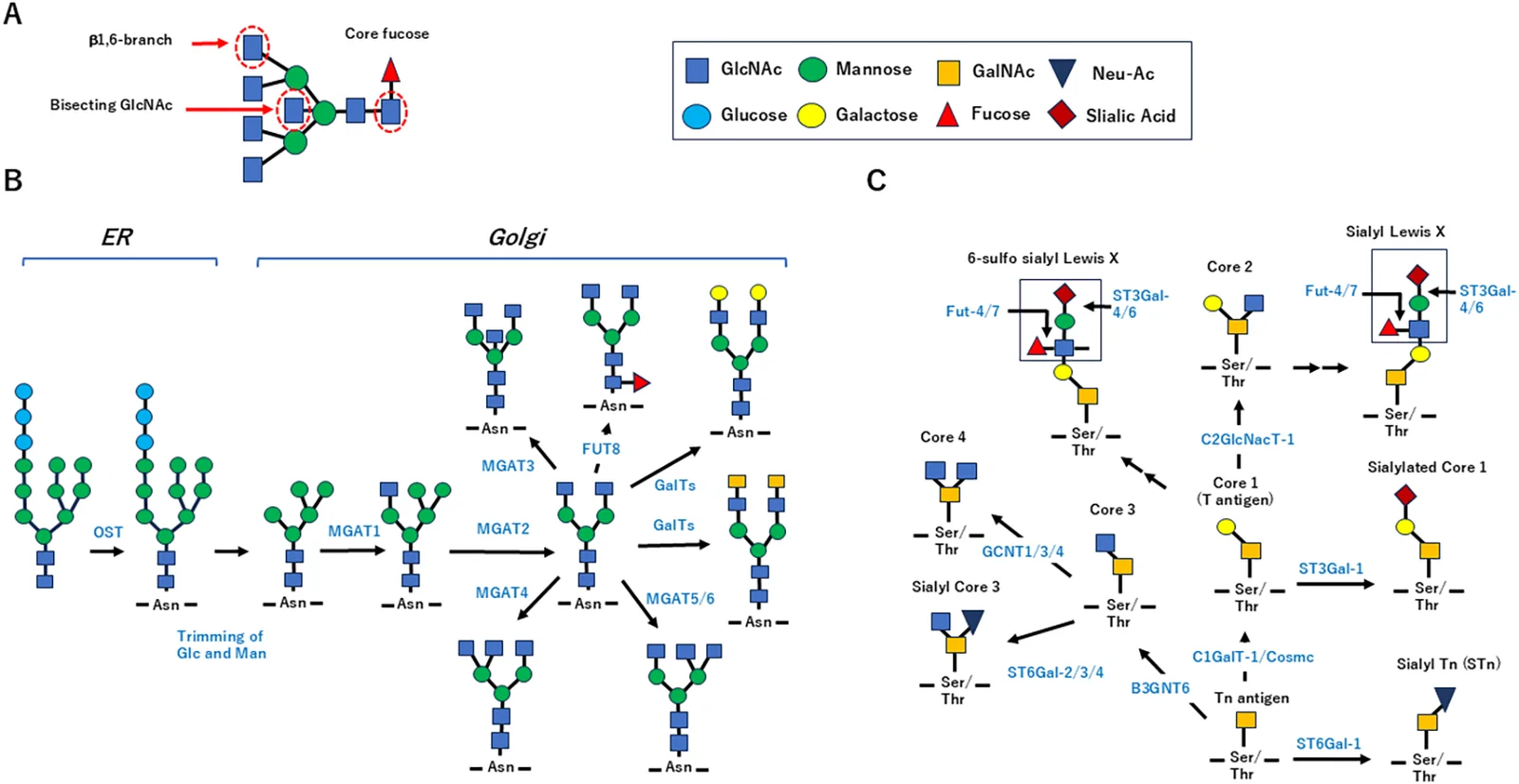
Schematic diagram of N-glycan branches and enzymes responsible for their synthesis. **(A)** Structure of N-glycan branches. All mammalian N-glycans have the common core pentasaccharide and multiple branches form on this core structure. **(B)** Schematic drawing of N-glycan biosynthesis in the ER and Golgi apparatus. A biantennary GlcNAc-terminated N-glycan is the common substrate for many glycosyl transferases, including branching enzymes. Notably, prior formation of one branch inhibits the formation of another branch, indicating that the reaction order of glycosyltransferases is important for shaping appropriate N-glycan structures in cells. GlcNAc, N-acetylglucosamine; OST, oligosaccharyltransferase; GalNAc, N-acetylgalactosamine; MGAT, β-1,6-N-acetylglucosaminyltransferase; FUT, α1–3 fucosyltransferase; GalT, α1–4 galactosyltransferase **(C)** Scheme of the mucin-type O-glycosylation and *Sialylation* pathway. All of the GTs involved in initiation and core extension are mentioned together with the stereochemistry and glycosidic linkages. Neu-Ac, N-acetylneuraminic acid; B3GNT-6, β 1–3 N-acetylglucosaminyl transferase-6; C2 GlcNAcT-1, β1–6 N-acetylglucosaminyl transferase 1, C2 GCNT-1/3/4, β1–6 N-acetylglucosaminyl transferase 1/3/4; FUT-5/7, α1–3 fucosyltransferase 5/7; ST3Gal-1, ST3 β -galactoside α 2–3 sialyltransferase -1; ST6Gal-1/2/3/4; ST6 β -galactoside a 2–3 sialyltransferase -1/2/3/4.

Glycosylation is one of the most common post-translational modifications of proteins and plays an important role in a variety of biological functions, including protein stability and effector function, intercellular interactions, signal transduction, and cellular immunogenicity. Protein glycosylation normally occurs in the endoplasmic reticulum (ER) and Golgi apparatus, although it can also occur in the cytoplasm and nucleus. Two main types of protein glycosylation are present in humans: N-linked and O-linked glycosylation. N-linked glycosylation refers to the attachment of glycans to the nitrogen atom of an asparagine residue in a protein and plays a crucial role in protein localization, secretion, immunogenicity, and immune response. O-linked glycosylation involves the attachment of glycans to the oxygen atom of serine or threonine and contributes to cell-cell interactions, signal transduction, virus infection, and other biological processes (**Figure 2**) (21).

#### Glycan binding proteins, modifiers in glycan-dependent cell function

Glycan-binding proteins represent a diverse group of molecules that recognize and interact with glycan structures, thereby mediating immune functions such as cell signaling and pathogen recognition (22, 23). In the mammalian immune system, three main families of glycan-binding proteins have been identified: galectins, C-type lectins, and sialic acid-binding immunoglobulin-like lectins (Siglecs). All of these lectins have at least one carbohydrate-recognition domain. Galectins are normally soluble proteins found in the extracellular milieu, while C-type lectins and Siglecs are typically membrane-bound. Galectins are expressed by all immune cells, and their expression is upregulated in activated T and B cells, inflammatory macrophages, natural killer cells, and regulatory T cells. There are three different forms of galectin structures: dimeric, tandem or chimera. Dimeric galectins are homodimers, consisting of two identical galectin subunits that have associated with one another. The galectins that fall under this category are galectin-1, -2, -5, -7, -10, -11, -14 and -15. Tandem galectins are considered intrinsically divalent and include galectin-4, -6, -8, -9 and -12. The final galectin is galectin-3 which is the only galectin found in the chimera category in vertebrates. Galectin-3 exist in monomeric form or can associate into multivalent complexes up to a pentameric form. Galectins recognize the disaccharide N-acetyllactosamine (LacNAc), composed of galactose and N-acetylglucosamine, which is present in complex N-glycans and O-glycans. C-type lectins contain one or more carbohydrate-recognition domains and are expressed in antigen-presenting cells (APCs), including macrophages and dendritic cells (DCs). These lectins recognize glycans containing mannose, fucose, N-acetylgalactosamine (GalNAc), or N-acetylglucosamine (GlcNAc). Siglecs are type I membrane proteins displaying an amino-terminal V-set immunoglobulin domain and recognize sialic acid-containing glycans. They are expressed in neutrophils, monocytes, B cells, NK cells, DCs, eosinophils, and basophils, and play a fundamental role in distinguishing self and non-self.

#### N-linked glycosylation pathway in cells

The core of N-linked glycans consists of two consecutive GlcNAc residues and three mannoses residues, which can be further extended and modified by various glycosyltransferases and glycosidases to generate oligomannose, complex, or hybrid N-glycans (**Figures 2A, B**). UDP-GlcNAc is one of the essential metabolites fueling N-glycosylation and is a crucial regulatory factor. The hexosamine biosynthetic pathway contributes to glycan biosynthesis by converting glucose to UDP-GlcNAc through a six-step process that shares the first two steps with glycolysis (**Figure 1B**). Briefly, glucose is phosphorylated by hexokinase to produce glucose-6-phosphate, which is subsequently converted to fructose-6-phosphate by glucose-6-phosphate isomerase. Fructose-6-phosphate is then converted to glucosamine-6-phosphate by the rate-limiting enzyme: glutamine fructose-6-phosphate amidotransferase (GFAT), which ultimately contributes to UDP-GlcNAc synthesis. UDP-GlcNAc serves as a substrate for the Golgi β-1,6,-N-acetylglucosaminyltransferase (MGAT) family of enzymes, including MGAT1, 2, 4 and 5 (**Figure 2B**). These enzymes regulate N-glycans branching, with the extent of branching depending on the metabolic flux through MGAT enzymes. The relative activity of each enzyme and the concentration of UDP-GlcNAc determines this flux. During N-glycan biosynthesis, early glycan transfer steps are obligatory; however, competition between two or more transferases occurs during later processing steps. For example, MGAT3 transfers a bisecting GlcNAc residue to the central mannose residue of the trimannosyl core structure of N-glycan, whereas MGAT5 transfers β6-linked GlcNAc residue to one of the outer mannose residues of the mannosyl core structure (**Figure 2A**). Once the bisecting GlcNAc is attached by MGAT3, further GlcNAc transfer by MGAT5 is markedly hindered. Similarly, MGAT1 has a higher affinity for UDP-GlcNAc than MGAT5; therefore, increased MGAT1 expression can inhibit further N-glycan branching by limiting UDP-GlcNAc availability to MGAT5. In addition to glycosyltransferase activity, substrate availability is a critical factor for successful N-glycan branching. The hexosamine biosynthetic pathway is the main source of UDP-GlcNAc, which is required for N-glycan branching. UDP-GlcNAc synthesis by hexosamine biosynthetic pathway may directly compete with glycolysis and glutaminolysis for fructose-6-phosphate and glutamine, respectively. Mannose is another key monosaccharide in N-glycan synthesis (**Figure 1B**). The N-glycan core requires nine mannose residues to form a single N-glycan. GDP-mannose can be generated from glucose or mannose via mannose-6-phasphate, a key precursor metabolite. This phosphorylated mannose is a key intermediate in synthesizing glycans and can be converted into glycan structures. Fucose can be derived from different sources. In *de novo* fucose synthesis, mannose or glucose are used as precursors, with GDP- mannose being converted to GDP-fucose through various intermediary species.

#### N-glycosylation in T cell biology

**Figure 3:**
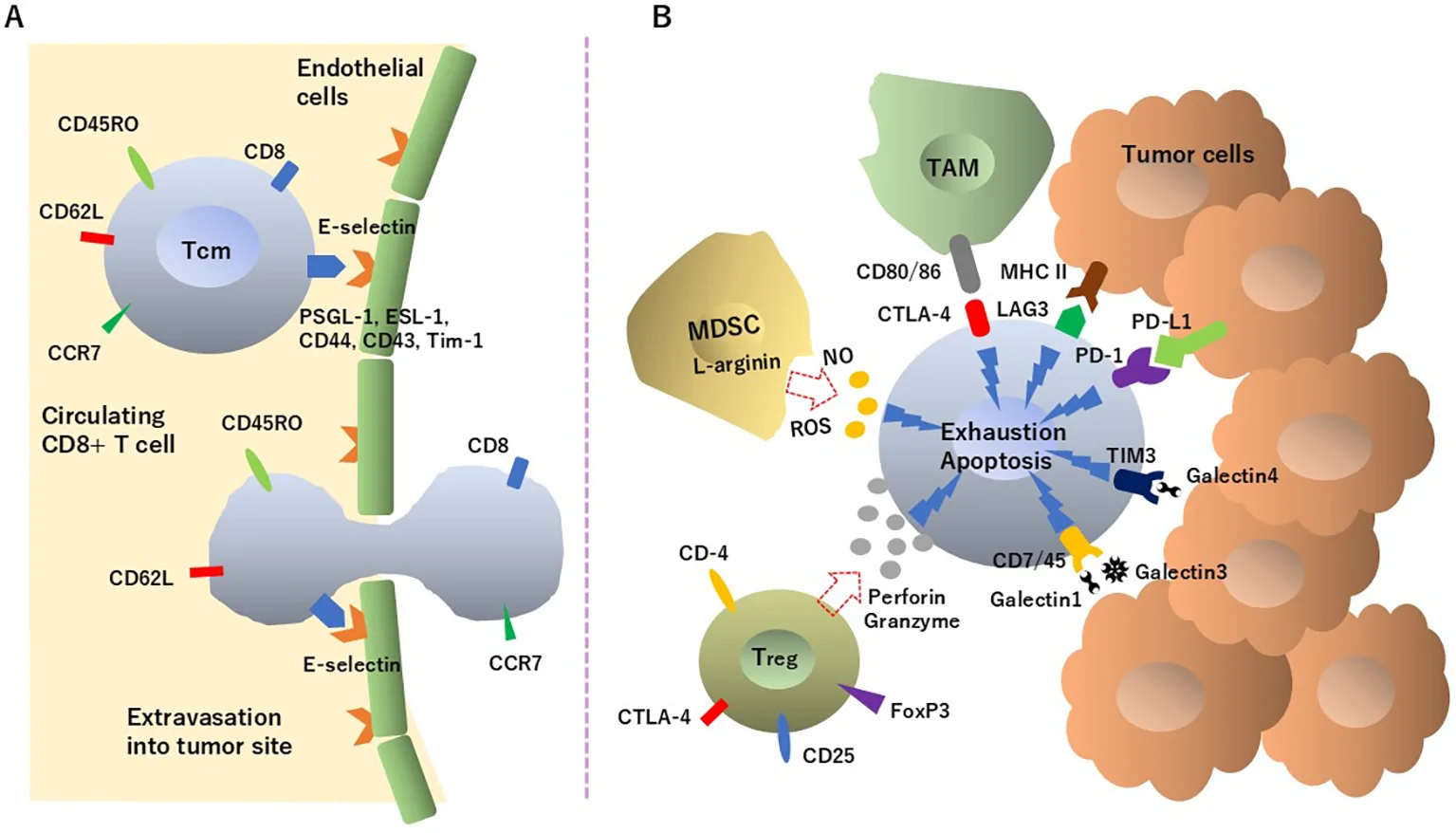
Immunosuppressive nature of the tumor microenvironment. **(A)** Trafficking of T cells into non-lymphoid tissue. Effector and memory T cells synthesize core 2 O-glycans on surface proteins including PSGL-1, ESL-1, CD44, CD43 and Tim1. These modified proteins function as P- and/or E-selectin ligands that facilitate capture of these T cells on activated vascular endothelium to initiate extravasation into non-lymphoid tissues. **(B)** Immunosuppressive nature of the tumor microenvironment. Immunosuppressive cells in the tumor microenvironment are tumor-associated macrophages (TAM), myeloid-derived suppressor cells (MDSC), and regulatory T cells (Treg). Under homeostatic conditions, galectins are the major immune regulators of T cells. T cell exhaustion and apoptosis are induced by the progressive acquisition of inhibitory receptor signaling pathways, including PD-1 bound to PD-L1, LAG-3 bound to MHC II, CTLA4 bound to CD80/86, TIM3 bound to galectin-4/9 and CD7/45 bound to galectin-1/3. T cell apoptosis is also induced by perforin and granzyme secreted by Tregs and by NO and ROS secreted by MDSCs.

T cells play a central role in the adaptive immune system, and their function depends on intra- and intercellular communication mediated by N-glycans and their corresponding binding partners (24) (**Figure 3**). Under homeostatic conditions, galectins act as major immune regulators of T cells. Galectins are soluble proteins that are widely expressed in various cell types and bind to cell surface glycoproteins and form galectin lattices, depending on the glycosylated sites. The minimal binding structure for galectins is LacNAc, and binding avidity for individual glycoproteins increases in proportion to the degree of LacNAc content within the attached N-glycans. Galectin -1 and Galectin-3 induce T cell apoptosis by binding to branched N-glycans containing the LacNAc motif found on their T cell counter-receptors such as CD7 and CD45. Galectin-9, unlike other galectin members with affinity to many cellular receptors, binds specifically to T cell immunoglobulin mucin 3 (TIM-3) expressed on T cells and induces apoptosis (25). Thus, the galectin-mediated T cell suppression is one of the mechanisms by which tumor cells escape immune attacks or even regulate immune responses.

IL-2 and IL-7 can modulate N-glycan branching in T cells. Cytokine-mediated physiological conditions alter N-glycosylation through modulation of glycosyltransferase activity (26). IL-2 and IL-7 reduce N-glycan branching in resting T cells, thereby promoting T-cell growth through enhanced TCR signaling, whereas they markedly increase N-glycan branching in activated T cells, thereby inducing growth arrest of T cell blasts. In resting T cells, IL-2 induces upregulation of MGAT1, which catalyzes the biosynthesis of mono-branched N-glycans, while simultaneously decreasing MGAT5 expression. Owing to its higher avidity for UDP-GlcNAc compared with MGAT4 and MGAT5, MGAT1 sequesters UDP-GlcNAc from distal MGAT4 and MGAT5, thereby reducing branching when UDP-GlcNAc is limiting. Conversely, in activated T cells, TCR signaling increases MGAT5 and UDP-GlcNAc, thereby exploiting IL-2 induced upregulation of MGAT1, resulting in increased N-glycan branching (26, 27). Consistently, a deficiency of MGAT5 lowers T cell activation thresholds by directly enhancing TCR clustering (28), while MGAT5-mediated increases in GlcNAc increase the binding to its ligand galectin 3, resulting in T cell apoptosis. IL-10 also induces expression of MGAT5 in T cells, which increases N-glycan branching on T cells. Eventually, the increased N-glycosylation promotes the formation of galectin-3-mediated membrane lattice and decreases antigen sensitivity (29).

#### N-glycosylation in Treg biology

Regulatory T cells (Tregs) are essential for the maintenance of immune tolerance and preventing autoimmunity (30, 31). Tregs represent a subset of CD4+T cells characterized by high expression of CD4, CD25, and the Forkhead box P3 transcription factor (FOXP3), along with low expression of the IL-7 receptor a-chain (CD127). Thymus-derived Tregs (rTreg) or natural Tregs (nTreg) require IL-2 for survival and maintenance of their suppressive ability and to recognize self-antigens. Another subset of Tregs, peripheral Treg (pTreg) or inducible Treg (iTreg), are differentiated by TGF-β signaling. With IL-2 exposure to peripheral tissues, they recognize foreign antigens. In the thymus, Tregs display higher levels of complex N-glycans, correlated with an increased suppressive ability (32). Several glycan-binding proteins, such as galectins and siglecs, are particularly important for Tregs biological function (30). Galectin-1 promotes the expansion of IL-10-producing Tregs, reinforcing their role in maintaining immune homeostasis. Galectin-3 inhibits endocytosis of surface receptors such as the immunosuppressive CTLA-4 by binding to complex branched N-glycans. Thus, increased retention of CTLA-4 on Tregs inhibits their cell differentiation and growth. Galectin-9 promotes Treg proliferation and stability by increasing FOXP3 expression through binding to CD44 and subsequently activating TGF-β signaling.

#### N-glycosylation in macrophage biology

Classically activated M1 macrophages typically exert anti-cancer function through direct, and antibody-dependent cell-mediated cytotoxicity (ADCC) to kill tumor cells. Alternatively activated M2 macrophages promote the occurrence and metastasis of cancer cells, inhibit T cell-mediated anticancer immune response, and promote tumor angiogenesis, leading to tumor progression. Galectins also participate in macrophage-mediated immunomodulation. Macrophages are activated to differentiate into the M2 profile through binding of N-glycosylated CD98 membrane receptor to galectin-1 and -3 (33).

#### N-glycosylation in cancer cell biology

One of the characteristics of tumors is the increased branching of N-glycans, regulated by MGAT expression levels, and is involved in regulating the interaction between tumor cells and the matrix and in promoting the migration of tumor cells (34, 35). Most receptors on the cell surface, including epidermal growth factor receptor (EGFR), insulin-like growth factor receptor (IGFR), basic FGF receptor (bFGFR), and transforming growth factor β receptor (TGFβR), are N-glycosylated. N-glycans are required for ligand binding. Intracellular galectins can enhance oncogenic signals and reduce apoptosis, thereby promoting cancer transformation and proliferation (36). Increased MGAT5 expression leads to elevated levels of complex β1-6-branched N-glycan, such as elongated N-glycan of EGFR, in tumors resulting in a loss of contact inhibition and increased cell motility, leading to cancer metastasis. The downregulation of MGAT5 decreases metastasis (37) and leads to reduced responsiveness to EGF, IGF, and PDGF than that in wild-type cells (38, 39). Thus, the inhibition of MGAT5 is useful in the treatment of malignancies by targeting its role in metastasis. On the contrary, increased expression of MGAT3 increases a bisecting GlcNAc, resulting in the suppression of further elongation of N-glycans. The decrease in elongated N-glycan reduces the ability of EGF to bind to its receptor, which inhibits its downstream signaling, including EGFR mediated Erk phosphorylation. Increased expression of MGAT3 also results in resistance of E-cadherin to proteolysis, and retention of E-cadherin at the cell-cell borders, leading to enhancement of cell-cell adhesion (40). Thus, increased expression of MGAT3 inhibits cancer metastasis and its activation is useful for suppressing cancer growth.

Integrins constitute another important receptor family. Integrin engagement during cell adhesion leads to intracellular phosphorylation of focal adhesion kinase (FAK), thereby regulating gene expression, cell growth, differentiation, and survival from apoptosis. N-glycans are essential for functional α5β1-integrin activity (41, 42). Increased β1,6 branched N-glycans on α5β1-integrin promote malignant tumor invasion and metastasis. Consistently, cell migration toward the extracellular matrix (ECM) and invasion are substantially stimulated in cells in which the expression of MGAT5 is induced. In fact, increased β1,6 branched N-glycans in tumor cells and cells over-expressing MGAT5 inhibit the clustering of α5β1-integrin and organization of F-actin into extended microfilaments in cells. Thus, modified N-glycans affect protein folding and stability, and have the ability to interfere with receptor-ligand interactions, and consequently, regulate cell growth, migration, differentiation, and cancer invasion.

#### O-linked glycosylation pathway in cells

GalNAc constitutes one of the 10 essential monosaccharides that, when activated as a sugar nucleotide (UDP-GalNAc), contribute to the construction of the human glycome (43). The addition of GalNAc onto Ser/Thr residues, and potentially Tyr, defines one of the most important, abundant, and complex regulated types of protein O-glycosylation pathways present in eukaryotes, known as GalNAc- or mucin-type O-glycosylation (**Figure 2C**). The O-glycosylation pathway is mediated by a large family of GalNAc-transferases (GALTs) that catalyze the sequential addition of monosaccharides in a stepwise manner. After the synthesis of the Tn antigen, O-glycans undergo diverse modifications; however, the most common forms contain one of four core structures that branch from the initial GalNac residue. Among these, core 1 and 2 are implicated in various immunological processes, including T-cell contraction through apoptosis, antibody function, antigen receptor activation, and cell adhesion and trafficking (44). In contrast, core 3 and 4 structures are less prevalent and there is no clear role for these O-glycans in immunity. A core 1 O-glycan (T antigen) is generated by core 1 β1-3-galactosyl transferase (C1GalT-1) through the addition of galactose to the initial GalNac residue via a β1–3 linkage. Core 1 structures may subsequently undergo extension and capping or serve as substrates for synthesis of core 2 O-glycans. The extension of core 1 and 2 structures is catalyzed by N-acetylglucosaminyltransferases and galactosyltransferases, which add repeated units of N-acetyllactosamine to the galactose of core 1 or the GlcNAc of core 2. Poly-N-acetyllactosamine repeats on core 2 O-glycans serve as acceptor sites for further modifications, including the addition of the tetrasaccharide Lewis X, critical for selectin ligand formation, and the addition of terminal sialic acids and fucoses, critical for generating the functional binding motif for all selectins.

#### O-linked glycosylation in T cell biology

Among the diverse roles of O-glycans in regulating T-cell biology, one of the most well-characterized role for these post-translational modifications is for capturing circulating T cells on vascular endothelium and allowing them to infiltrate lymphoid and non-lymphoid tissues (**Figure 3A**) (44). Selectins, including E-selectin (CD62E), P-selectin (CD62P), and L-selectin (CD62L), are members of the C-type lectin domain containing proteins that bind to specific carbohydrate structures decorated with O-glycans in a calcium-dependent manner. E-selectin is expressed on endothelial cells, whereas P-selectin (CD62P) is stored in α-granules of platelets and endothelial cells. Following infection or tissue injury, P- and E-selectin are expressed by the vascular endothelium in response to inflammatory cytokines such as IL-1 and TNF-α. In contrast, L-selectin is constitutively expressed on many leukocytes. Selectin ligands can be divided into two functional categories: those involved in steady-state lymphocyte homing to secondary lymphoid organs and those responsible for leukocytes recruitment during inflammation or tissue injury. Lymphocyte homing to lymph nodes is primarily mediated by L-selectin ligands, such as peripheral node addressin (PNAd), which are constitutively expressed by endothelial cells, while leukocyte homing into non-lymphoid tissue is mediated largely by P- and E-selectin ligands, such as P-selectin glycoprotein ligand-1 (PSGL-1) and the T cell immunoglobulin and mucin domain glycoprotein 1 (TIM1), expressed by CD4+ T cells. Several other leukocyte-expressed proteins besides PSGL-1 can bind to E-selectin, including E-selectin ligand-1 (ESL-1), CD44, and CD43. In all cases, the unmodified selectin ligand is unable to bind to selectins, rather this interaction is dependent on the presence of specific O-linked glycosylation modifications. Naïve T cells do not synthesize core 2 O-glycans and fail to bind to P-and E selectin, which excludes them from entering non-lymphoid tissues. Instead, naïve T cells express L-selectin, which serves as the receptor for ligands such as PNAd expressing core 1 O-glycans in the endothelial venules of lymph nodes. Following stimulation of the T cell receptor, effector T cells increase core 2 O-glycans synthesis, which transforms surface proteins into P- and E-selectin ligands to direct extravasation across activated vascular endothelium and into non-lymphoid tissues.

#### O-linked glycosylation in cancer cell biology

A prominent characteristic of cancer cells is the increased presence of truncated and aberrant O-glycans caused by overexpression of ST6GalNAc-I (45). ST6GalNAc-I converts Tn into sialyl-TN (STn), inhibiting the extension upon GalNAc residues. The altered glycosylation with unique structure in tumors is known as tumor-associated carbohydrate antigens (TACAs). TACAs, the most relevant ones being the Tn, T, and STn antigens, are found in clinical specimens of different types of malignant tumors (**Figure 2C**). The altered O-glycosylation plays crucial roles in facilitating tumor formation, progression, and metastasis (34). Moreover, the binding of tumor-associated STn to lectins expressed on immune cells inhibits dendritic cell maturation and impairs antigen presentation, thereby suppressing T-cell stimulation and activation and facilitating immune escape.

#### Sialylation pathway in cells

Sialylation, the process of adding sialic acid residues at the termini of glycans, is a crucial modification that plays diverse roles in cell recognition, adhesion, and intercellular signal transduction through interactions with Siglecs (46–48). Siglecs typically transduce signals via immunoreceptor tyrosine-based inhibition motif (ITIM)-mediated signaling cascades. Phosphorylated ITIMs recruit the SHIP1 and SHIP2 tyrosine phosphatases, which subsequently modulate downstream signaling upon activation.

#### Sialylation in immune cell biology

DCs are antigen-presenting cells with the ability to take up antigens in the periphery and expose them to lymphocytes, thus bridging the gap between innate and adaptive immune response (49). Sialoglycan-covered antigens are mainly recognized by Siglecs on DCs that signal through immune inhibitory cytoplasmic tails. Thus, over-expressed Siglec ligands on tumor cells are involved in hampering DC-induced T cell-mediated killing (50).

NK cells are involved in cell-mediated cytotoxicity and the secretion of proinflammatory cytokines (51). FcgRIIIa (CD16a) is the most abundantly expressed activating receptor on circulating NK cells and mediates ADCC. Siglec-7/9 expressed on NK cells, as well as Siglec-9 on tumor-infiltrating CD8 T cells, reduce tumor cytotoxicity through interactions with tumor-associated sialoglycans (48). Thus, the sialoglycan-Siglec axis contributes to the formation of an immunosuppressive TME.

Sialylation affects M1/M2 macrophage polarization. M1 macrophages exhibit downregulated ST6GAL1 expression, resulting in reduced a2,6-sialic acid, whereas M2 macrophages exhibit higher ST6GAL1 production than M1 macrophages, resulting in the gain of a2,6-sialic acid. ST6GAL1-mediated a2,6-sialylation regulates TNF-a death receptor 1 activity; increased ST6GAL1 activity promotes M1 macrophages apoptosis and M2 macrophages survival. Elevated levels of sialic acid are considered a hallmark of tumor cells and directly affect the interaction between tumor cells and the TME, especially the interaction with Siglecs in the regulation of immune cell function (33). Over-expressed Siglec ligands on tumor cells are involved in the differentiation of tumor-infiltrating monocytes to monocyte-derived macrophages, tumor-associated macrophages (TAMs) and myeloid-derived suppressor cells through Siglec-9/10/15 on monocytes (50). Siglec-9 in primary monocytes and macrophages induces TAM to promote tumor progression through binding to abnormally sialylated MUC1 glycan in the tumor cells. Siglec-10 in macrophages blocks the cytoskeletal rearrangement required for cellular engulfment by macrophages by binding to CD24 on tumor cells. Siglec-15 in TAM triggers the production of immunosuppressive TGF-β by binding to truncated STn on tumor cells. Blocking of Siglecs-9/10/15 with antibody enhances the phagocytosis of tumor cells by macrophages (52). Conversely, administration of sialidase to induce desialylation polarizes TAMs to an immune-promoting M1 macrophage phenotype.

#### Sialylation in cancer cell biology

α2–6 sialylation, particularly mediated by ST6GAL1, exerts a strong tumor-promoting role in many tumor cells (47). Mechanistically, this is linked to the modification of receptors on the cell surface that regulate cell growth and apoptosis. For example, TNFR1 triggers caspase-mediated apoptosis upon binding to TNF. ST6GAL1-mediated α2–6 sialylation of TNFR1 promotes tumor cell survival by preventing TNF binding and subsequent internalization. FAS and PDGF receptors also undergo a similar type of regulation through ST6GAL-1. Sialic acids also contribute to invasion and metastasis of cancer cells by promoting cell detachment and facilitating tumor cell migration through classical rolling mediated by adhesion receptor interactions between selectins and their sialyl-Lewis antigen ligands.

#### Fucosylation pathway in cells

Fucosylation, particularly core fucosylation, represents one of the most widespread tumor-related changes in the N-glycans (53). α-1,6-Fucosyltransferase (FUT8), the sole known enzyme responsible for generating an α-1,6-fucosylated structure at the core residue of N-glycan, regulates T-cell function (54). The TCR receptor requires FUT8-mediated core-fucosylated N-glycans for proper activation. Fucosylation also affects M1/M2 macrophage polarization. M1 macrophages express 5–10 times higher levels of fucosyltransferases (FUT1, 3, 7, 9) than monocytes. Inhibition of FUT1, 2 promotes a shift in M1 macrophages differentiation toward M2 macrophages.

Conversely, core fucosylation is required for the expression of programmed cell death receptor 1 (PD-1), which is responsible for attenuating TCR signaling. Hyper-core-fucosylation induces upregulation of PD-1 expression, thereby impairing T-cell function. In contrast, inhibition of FUT8 expression markedly diminishes PD-1 core fucosylation, correlating with decreased PD-1 expression and augmented T cell activation.

## Glycosylation and immune cells in cancer therapy

### Glycosylation and immune checkpoint inhibitors in cancer therapies

Tumors evade immune surveillance to survive within the host. They achieve this through multiple mechanisms, one of which is the promotion of T-cell exhaustion (1) (**Figure 3B**). Exhausted T cells exhibit reduced survival capacity, proliferation, and cytokine secretion. T-cell dysfunction is characterized by the progressive acquisition of inhibitory receptors, including PD-1, lymphocyte activation gene-3 (LAG-3), cytotoxic T-lymphocyte-associated protein 4 (CTLA4), and T cell immunoglobulin and mucin domain-containing protein 3 (TIM4) (2, 3). These inhibitory surface molecules are defined as immune checkpoints.

Engagement of PD-1 on T cells with PD-L1 expressed on tumor cells causes the recruitment of inhibitory phosphatase such as SHP2 to the intracellular domain of PD-1. Bound PD-1 directly inhibits TCR-CD28 signaling through Ras-MAPK and PI3K signaling to impair T cell proliferation and cytokine secretion (55). Blockade of inhibitory receptors using antibodies against PD-1 or PD-L1, known as checkpoint inhibition therapy, can sustain effective T cell immunity to cancers (56). However, clinical trials demonstrated that many patients eventually develop resistance to checkpoint inhibition therapy. Although genetic, transcriptional, and post-transcriptional regulation have been studied, accumulating evidence indicates that post-translational modifications, particularly glycosylation, are critical regulators of PD-L1/PD-1 function (57). N-glycosylated PD-L1 at 4 asparagine residues in the extracellular domain on tumor cells inhibits T cell activity through PD-L1/PD-1 interaction in the tumor microenvironment, whereas non-glycosylated PD-L1 reduces immunosuppressive activity owing to its inability to bind to PD-1 (58). N-glycosylated PD-1 at 4 asparagine residues in the extracellular domain on tumor-specific T cells maintain PD-1 protein stability and cell surface localization and mediates PD-L1/PD-1 interaction (59). Thus, N-linked glycosylation of PD-L1 and PD-1 plays a pivotal role in their stability and interaction, ultimately promoting PD-L1/PD-1-mediated immune evasion (60, 61).

CTLA-4 and CD28 are homologous receptors expressed on T cells that exert opposing functions in T-cell activation. Binding of CD28 to its ligands CD80 or CD86 on tumor cells activates IL-2 transcription through adaptor proteins and several kinases, thereby promoting T-cell proliferation. In contrast, CTLA-4 exhibits significantly higher affinity and avidity for CD80 and CD86 than CD28. Bound CTLA-4 activates intracellular signaling pathways that ultimately result in IL-2 downregulation, T-cell apoptosis, and anergy (55). CTLA-4 is a homodimeric inhibitory receptor. Its homodimer binds to homodimeric CD80 on tumor cells, allowing CTLA-4 to sequester CD80 ligands and antagonize CD28-dependent co-stimulation (62). In conjunction with disulfide bond formation, N-glycosylation of CTLA-4 at two asparagine residues in the extracellular domain is required for proper dimerization (63). Blockade of CTLA-4 using antibodies against CTLA-4 improves overall survival and progression-free survival in clinical studies of patients with advanced melanoma. Nonetheless, there are still some treated patients who do not respond. Currently, some preclinical trials investigating the use of anti-CTLA-4 with other immune checkpoint inhibitors are in progress to achieve a more potent therapeutic effect.

Given that N-linked glycosylation of PD-L1 and PD-1 is essential for immune evasion, multiple small-molecule agents targeting N-glycosylation have been developed and evaluated in cancer models. 2-DG reduces cell-surface PD-L1 expression by disrupting N-glycosylation. Combination therapy with 2-DG and the EGFR inhibitor gefitinib inhibits PD-L1-mediated immunosuppression (64). *In vivo*, combining D-mannose with anti-PD-1 significantly inhibits tumor growth in triple negative breast cancer models (65). Metformin decreases the stability and membrane localization of PD-L1 by disrupting N-linked glycosylation. Combining metformin with anti-CTLA-4 significantly improves tumor size and cytotoxic T cells activity in triple negative breast cancer models (66). Thus, the implication of metabolic targeting agents on anticancer treatment proves to be effective in combination with other immunotherapies through inhibiting N-glycosylation of immune inhibitory receptors rather than glycolytic pathways.

As mentioned, N-linked glycans in the extracellular domain of PD-L1 hinder the recognition and binding of therapeutic molecules such as anti-PD-1 or anti-PD-L1 antibodies. Therefore, the development of monoclonal antibodies (mAbs) that specifically target N-linked glycosylated PD-L1 or antibody-drug conjugates with de-glycosylation capabilities has become a promising new therapeutic strategy. STIM108, an antibody which specifically recognizes highly N-linked PD-L1, inhibits PD-L1/PD-1 interaction and promotes cell-surface PD-L1 internalization and degradation (67). STIM108 exerts a potent cancer cell killing effect in combination with antimitotic drugs (67). Similar to PD-L1, N-glycan in the extracellular domain of PD-1 plays crucial roles in the binding of anti-PD-1 antibodies. STM418, an antibody specifically targeting glycosylated PD-1, exhibits higher binding affinity for PD-1, effectively hindering the PD-L1/PD-1 interaction and leading to potent anti- cancer immunity (59). Thus, targeting PD-L1 N-glycosylation and PD-1 N-glycosylation are promising strategies for improving the efficacy of immune therapy.

### Glycosylation and CAR-T cells in cancer therapy

**Figure 4:**
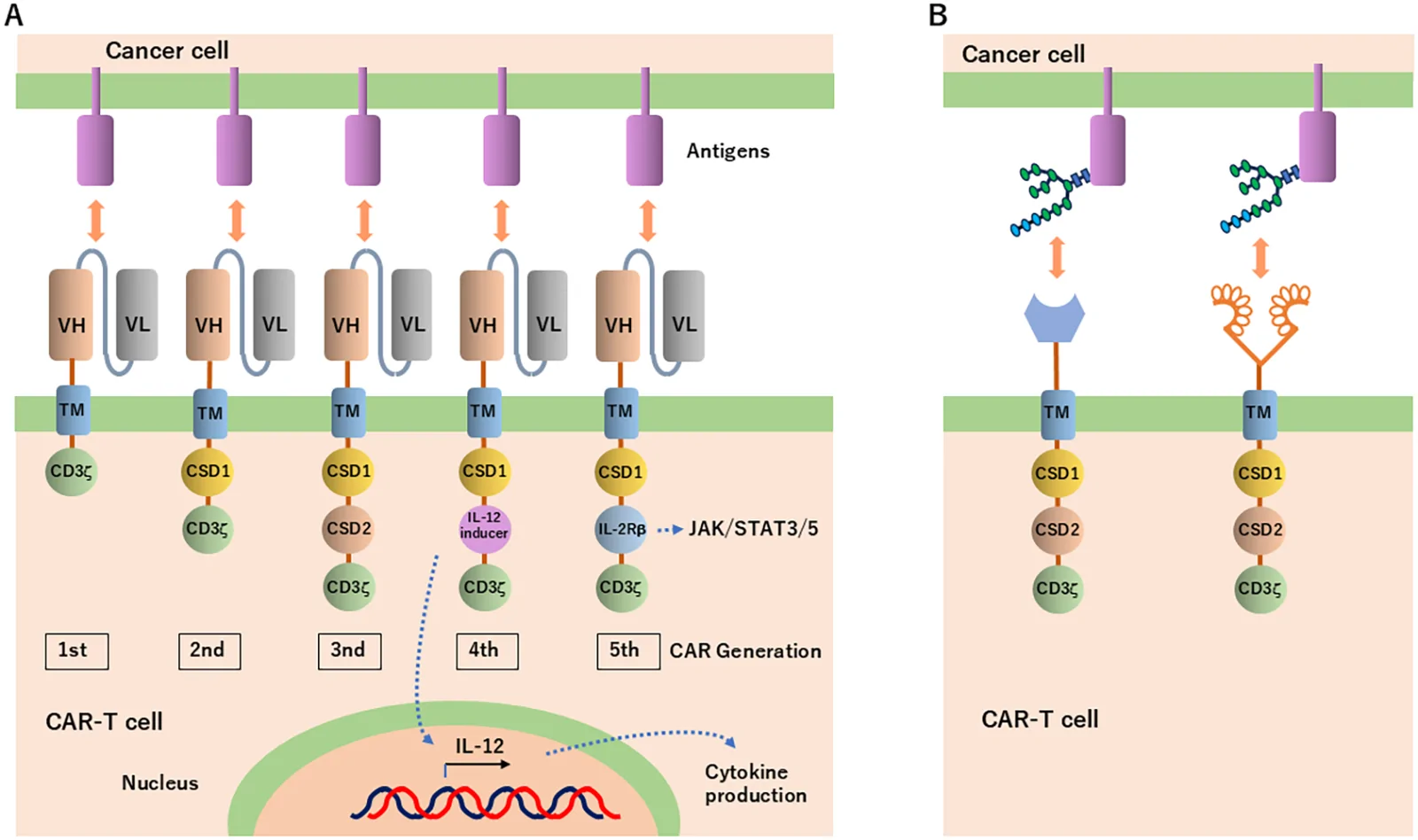
Structure and functionality of CARs. **(A)** Diagram of the five different generations of CAR targeting cancer antigens. A CAR construct is typically composed of four regions: an extracellular antigen-binding domain, commonly a single-chain variable fragment (scFv) fragment composed of variable light (VL) and heavy (VH) chains connected via a flexible linker sequence, an extracellular hinge region, a transmembrane domain (TMD) that anchors the CAR to the cell membrane and intracellular signaling module. CARs can be classified according to the complexity of their endodomain, being grouped into five different generations. First-generation CARs present a single activation signaling motif, derived from a CD3-z chain. Second-generation CARs include one co-stimulatory domain (CSD) 1, derived from the intracellular domain of CD28 or 4-1BB (CD137) and a CD3-z chain. Third-generation CARs, based on the second-generation CARs, contain additional CSD2 derived from OX40 (CD134), CD27, and ICOS. The fourth generation CARs, based on the second-generation CARs, contain a constitutive or inducible expression of cassette, leading to the transcription of a protein, such as IL-12. Fifth-generation CARs, also based on second-generation CARs, present intracellular domains of cytokine receptors, such as IL-2 receptor β-chain (IL-2Rβ) chain fragment, with a STAT3/5 binding motif. **(B)** Diagrams of CAR targeting glycans. CAR construct is composed of lectin extracellular domain (left), or smart anti-glycan reagent (SAGR; generated from lamprey antibodies) extracellular domain (right), instead of an extracellular antigen-binding domain, followed by the second-generation CARs.

Adoptive cell transfer therapy, particularly CAR-T cell therapy, represents a new stage in cancer immunotherapy. In CAR-T cell therapy, patient-derived T cells are genetically engineered *in vitro* to express CARs that function as navigation systems, enabling T cells to specifically recognize and efficiently eliminate cancer cells while enhancing the immune response (68). However, owing to the tumor immune escape mechanisms, the majority of patients fail to achieve sustained clinical benefit from immunotherapy. Therefore, exploring immune escape mechanisms and developing new strategies to overcome them are essential for improving immunotherapeutic efficacy. Two main approaches have been developed to optimize CAR-T therapy: the design of CAR structure and the intervention during CAR-T cell expansion stage (69). First, the CAR structure, including the antigen-binding domain, extracellular spacer, transmembrane domain, and intracellular signaling domain, is considered the core functional unit of the CAR-T cells (**Figure 4A**) (69, 70). The extracellular antigen-binding domain of CARs is derived from monoclonal antibody single-chain variable fragments (scFvs) (71). These scFvs are small, single-chained and designed to retain the affinity and specificity of the antibodies toward target epitopes, and consist of variable heavy (VH) and light (VL) chains connected by a flexible linker sequence. The extracellular domain is linked to the cytoplasmic signaling domain through the extracellular hinge and transmembrane domains. The cytoplasmic domain functions as the signal transduction region responsible for the triggering of T cell activation and regulating the magnitude and direction of cytotoxic effector responses. Upon antigen binding to the extracellular domain, T-cell activation is triggered through phosphorylation of ITAMs within the cytoplasmic CD3-z domain. The composition of the cytoplasmic domain can be manipulated to enhance a CAR’s specificity, potency and safety, giving rise to five different generations of CARs. First-generation CAR is characterized by a single activation cytoplasmic domain. Second- and third-generation CARs contain one and two co-stimulatory modules, respectively. Fourth-generation CAR bears additional immunomodulatory properties, by harboring a cytoplasmic module that sustains the release of transgenic cytokines. Fifth-generation CAR exhibits improved anticancer cytotoxic properties, owing to the addition of signaling subdomains of cytokine receptors that activate cytokine-dependent signaling pathways (72). Currently, most of CARs approved for clinical use are second-generation CAR.

A major limitation in the development of CAR-T cell therapies for solid cancers is the lack of tumor-specific targets with restricted expression on tumor cells. Effective protein targets for CAR-T cell therapies have been developed (73). Clinical trials of CAR-T cell therapies registered for the treatment of solid cancers include the receptor tyrosine kinase erbB-2 (HER2), Epidermal growth factor receptor (EGFR), Carcinoembryonic antigen (CEA), receptor tyrosine kinase-like orphan receptor 1 (ROR1), NKG2D ligands (NKG2DL2), B7-H3, also known as CD276, CD70, Claudins, and Mesothelin (72). However, a notable characteristic of malignant cells is the aberrant expression of cell-surface carbohydrates (74). These carbohydrates, TACAs, differ between healthy and malignant cells, and play important roles in facilitating tumor formation, progression, and metastasis. TACAs are specific and expressed on different cell surface molecules in a cancer-type specific manner. The most common abnormal glycosylations are O-glycan truncation, increased N-glycan branching, and changes in sialylation and fucosylation (75). The O-glycan truncation, also called cancer-associated O-glycans, includes Tn, STn and T (**Figure 2C**). Changes in O-glycosylation, either acting inside cells or on the cellular surface have a profound influence on many events in cancer development and progression, including signaling, stability and function of receptor proteins and adhesion to the matrix and to other cells (45). The increase in N-glycan branches is involved in regulating the interaction between tumor cells and the matrix and in promoting the migration of cancer cells, which is considered one of the characteristics of cancer (34). Elongated branching of N-glycans in the surface proteins on the cancer cells can impede the effectiveness of CAR-T cells targeting solid tumors by disrupting the formation of immune synapses, thereby diminishing transcriptional activation, reducing cytokine production, and lowering cytotoxicity. In contrast, targeted glycosylation can reshape the tumor microenvironment and promote the immune response, thereby improving the response to immunotherapy (45, 60, 76). The anticancer effect of CAR-T cells was significantly enhanced when the expression of N-glycosylation was weakened by the knockout of the glycosyltransferase MGAT5 in pancreatic cancer cells (77). Thus, the implication of abnormal glycosylation in cancer cells has recently attracted significant interest because of its potential as targets for immunotherapy.

Abnormal glycosylation of TACAs has emerged as a promising target for immunotherapy. However, carbohydrate antigens often exhibit low immunogenicity (78), posing an obstacle to T cell engagement. Despite this limitation, clinical trials of CAR-T cell therapies for TACAs registered for the treatment of various TACA-expressing solid cancers are underway (73). Heparan sulfate proteoglycans (HSPGs) are present on the ECM and the surface of many cellular types, especially highly abundant on tumor cells compared to healthy ones, and in malignant transformation. Heparan sulfate is mainly present on two membrane-anchored proteoglycans: glypicans (GPC1 to GPC6) and syndecans. Anti-GPC3 CAR-T cells show potent anticancer *in vivo* efficacy (79). Mucins are high-molecular weight and heavily glycosylated proteins that are the main component of the mucus. MUC1, a membrane-bound mucin, is closely associated with the development of cancers of glandular epithelial origin such as multiple adenocarcinomas. The most frequent aberrant glycosylation found in MUC1 are the Tn and the STn antigens. Anti-Tn and anti-STn antigen CAR-T cells display effective anti- cancer activity against leukemia and pancreatic cancer (80). Gangliosides, glycosphingolipids bound to the cell membrane and mostly expressed in neural tissue, facilitate cell-to-cell adhesion. GD2, a disialoganglioside, is exclusively upregulated on almost all neuroblastomas and melanomas and recognized as one of the top cancer antigen targets for immunotherapy by the National Cancer Institute (NCI). Anti-GD2 CAR-T cells are currently being developed to treat patients with high-grade glioma, osteosarcoma, and neuroblastoma, and many other sarcomas and solid cancers (81). Lewis blood group antigens are specific carbohydrate structures found on the surface of red blood cells. Blood-group-related TACAs include Lewis Y, X, and A. Lewis Y is aberrantly expressed on the surface of most epithelial-derived tumors. Anti-Lewis Y CAR-T cells were applied for leukemia and advanced solid cancers.

The generation of glycan-binding antibodies has also been hampered by significant obstacles. New approaches to use non-antibody glycan binders, such as lectins, have been developed (**Figure 4B**). Lectins are carbohydrate-specific proteins. Lectin-glycan interactions mediate various essential cellular processes in normal cells and are involved in many of the oncogenic activities of cancer-associated glycans (82). CAR that is guided by a lectin to a tumor *in vivo* has been developed (83). Alternatively, smart anti-glycan reagents (SAGRs) composed of highly specific anti-mammalian glycan antibodies fused with immunoglobulin chimeras are generated through a large-scale screening of lamprey lymphocyte cDNA libraries with unique glycan epitopes (84). Glycan-binding CAR-T cells disrupt the supportive stroma in pancreatic ductal adenocarcinoma as well as demonstrating potent anticancer activities in preclinical models (85). Thus, glycan-binding CAR-T cell approaches could overcome two of the primary obstacles to CAR-T cell therapy for solid cancers in general; lack of tumor-specific antigens and inability of CAR-T cells to penetrate the tumor microenvironment.

### Optimization of CAR-T cells in cancer therapy

In addition to structural design of CARs targeting cancer-specific antigens, interventions during the CAR-T cells expansion stage can significantly influence therapeutic efficacy. CAR-T cells exhibit strong proliferation and differentiation abilities in the culture stage (86). However, these activities are accompanied by competition between effector function and long persistence, which ultimately determines the final quality of the cell therapy after infusion into patients. The immunosuppressive nature of the TME represents an important factor (87). To improve the efficacy of cell immunotherapy and enhance the resistance of T cells to tumor cell immunosuppression, the *in vitro* culture condition and application of drugs of immune cells have been focused (88). With the discovery that T cells exhibiting a less differentiated phenotype, such as stem cell memory T cells, are correlated with improved clinical outcomes, many studies have worked to maintain this population in CAR-T cell products (89).

Standard *ex vivo* expansion procedures typically involve anti-CD3/CD28-activated T cells and are supplemented with exogenous IL-2. However, these procedures frequently accelerate terminal differentiation and senescence of T cells, as initial TCR stimulation, CAR tonic signaling, and cytokine exposure activate signaling transduction pathways that drive effector T cell differentiation. Therefore, improving CAR-T cell fitness requires supplementation with mediators that suppress cellular programs associated with terminal differentiation, exhaustion, and senescence (88). IL-2 is a widely used cytokine for T-cell culture because it promotes rapid T-cell proliferation *in vitro* but also evokes a switch from OXPHOS to glycolysis. It induces the formation of more effector T cells and reduces memory T cell population (90). Other cytokines, including IL-7, IL-15, and IL-21, mediate metabolic adaptation by boosting OXPHOS and inhibiting glycolysis (91). The combined use of IL-7 and IL-15 results in CAR-T cells with higher proportions of stem cell memory T cells and central memory T cells, and superior anticancer activity *in vivo* compared with CAR-T cells cultured with only IL-2 (92). IL-21 enriches a less differentiated phenotype of T cells. While IL-21 alone does not result in robust CAR-T cell proliferation, combination of IL-21 with other cytokines such as IL-7 and IL-15 or IL-2 improves CAR-T cell effector function (93).

Additional signaling pathways involved in T-cell activation have also been investigated. The PI3K/AKT/mTOR pathway promotes glycolytic metabolism and suppresses the transcription factor FOXO1, thereby facilitating T-cell proliferation and effector differentiation. Addition of Akt inhibitors during manufacture results in enrichment of CAR-T cells with central memory T phenotype and enhanced anti- cancer efficacy in different xenograft models, without compromising cell yields (94, 95). Addition of PI3K blockade also maintains a less differentiated phenotype with enhanced *in vivo* persistence and anticancer efficacy (96, 97). Notably, utilizing PI3K blockade has more feasibility to generate CAR-T cells with superior anti- cancer activity in xenograft models. Beyond targeting PI3K/AKT/mTOR pathway, blockade of IL-2-inducible T-cell kinase, GSK-3β, p38 kinase, or a Src-family kinase enriches stem cell memory T-cell populations by promoting gene expression programs associated with memory T-cell differentiation (98–101). These manufacturing strategies induce CAR-T cells enriched in central memory T subsets with lower expression of exhaustion markers including PD-1, TIM3, and LAG-3, thereby enhancing *in vivo* anticancer function in a xenograft cancer model, compared with non-treated CAR-T cells. In addition to signaling cascade blockade, other efforts to maintain minimally differentiated CAR-T cells include epigenetic interventions. The inhibition of a bromodomain and extra-terminal motif (BET) protein enriches CAR-T cells with stem cell memory T cell and central memory T cell phenotypes through down-regulating BATF gene expression in CD8+ T cells. Treated CAR-T cells inhibit T cell differentiation into effector phenotype, and exhibit superior *in vivo* persistence and anticancer effect (102).

**Figure 5:**
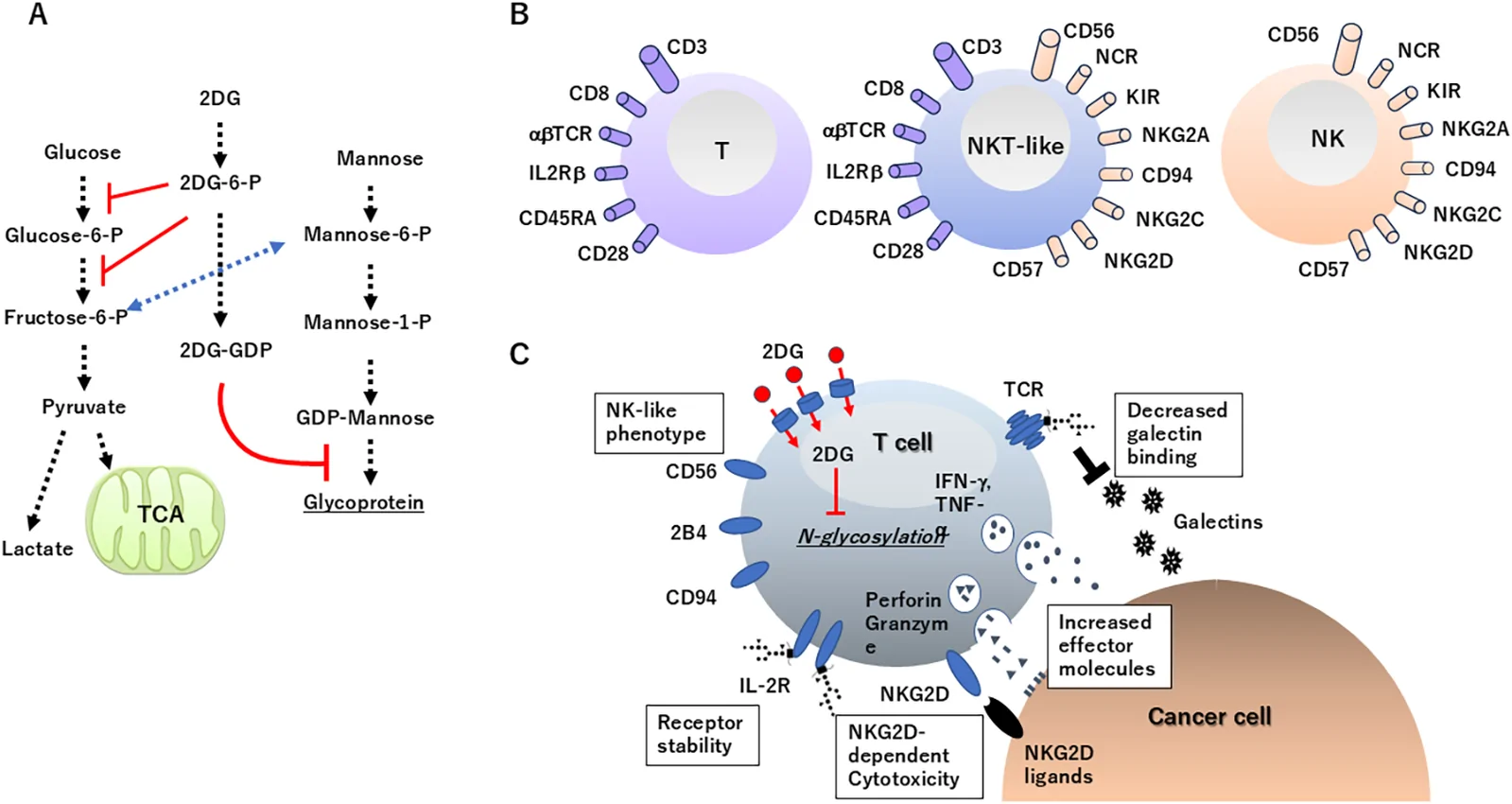
Promotion of anti-tumor immune response of T cells treated by 2DG. **(A)** Diagram of glycolysis and N-linked glycosylation pathways, with steps blocked by 2DG **(B)** NKT-like cells are T cells with the expression of the CD56, as a typical marker of NK cells. (Left) the receptor repertoire of T cells that are common to NKT-like cells, (Middle) the receptor repertoire of NKT-like cells that are common to T cells and NK cells, (Left) the receptor repertoire of NK cells that are common to NKT-like cells. T and NK cells are categorized as having an adaptive and innate immunity, respectively, whereas NKT-like cells share mechanisms from T and NK cells. The mature NKT-like cells (CD45RA and CD57) include a number of activating and inhibitory receptors (NCR, KIR, NKG2A/C/D, αβ TCR and IL-2Rβ). **(C)** Activation of T cells by 2DG-mediated blockage of N-glycosylation includes preferential targeting of NKG2D ligand-expressing tumor cells, increased secretion of TNF-α, IFN-γ, cytotoxic perforin and granzyme, surface stability of IL-2R with higher responsiveness to IL-2, and decreased interaction with immunosuppressive galectin released from tumor cells.

On the other hand, *ex vivo* priming of immune cells using metabolic targeting agents has been adopted in cell-based immunotherapy. Glycolysis supports the proliferation of effector T cells. Enforcing glycolytic metabolism inhibits the generation of early memory CD8+ T cells (103, 104), whereas inhibition of glycolysis leads to development of more stable memory CD8+ T cells. Metformin, a mitochondrial respiratory complex inhibitor, is commonly used in the treatment of type II diabetes, owing to its effect of reducing hepatic glucose production. A link between metformin usage and reduced risk of cancer in individuals with type II diabetes was established from retrospective epidemiological studies (105). These processes increase OXPHOS and appropriate inhibition of glycolysis *in vitro*, leading to a high percentage of stem cell memory T cells and central memory T cells, ultimately resulting in superior longevity and anti- cancer potential for CAR-T immunotherapy. 2-deoxy glucose (2DG) is a glucose analogue that has the 2-hydroxyl group replaced by hydrogen, so that it cannot undergo further glycolysis (**Figure 5A**). As such, it acts to competitively inhibit the production of glucose-6-phosphate from glucose at the phospho-glucoisomerase level. In parallel with the inhibition of glycolysis, 2DG is converted to 2-deoxy-D-mannose, and incorporated into the lipid-linked oligosaccharide precursor, thereby blocking N-glycan biosynthesis (106, 107) (**Figure 5A**). It has been reported that the *ex vivo* priming of mouse CD8+ T cells in the presence of glycolytic inhibitors, such as 2DG, favors the generation of long-lived memory T cells displaying superior anticancer activity once transferred *in vivo* in mouse xenograft models (108). Furthermore, our laboratory recently demonstrated the efficacy of 2DG as a blocker of N-glycosylation pathways in adoptive cancer immunotherapy (106, 109). Combining the knockout of MGAT5 with 2DG leads to loss of N-glycosylation in tumor cells and increases the anti- cancer efficacy of CAR-T cells (77). Collectively, the use of 2DG represents a promising strategy for overcoming the limitations of CAR-T cell therapy in the treatment of solid cancers.

### NK-like T cell in cancer therapy

Our laboratory has demonstrated that 2DG induces the differentiation of human peripheral T cells into NK-like T cells through inhibition of the N-glycosylation pathway (106, 109). Notably, the acquisition of NK cell properties by 2DG is upregulated by the transcriptional factors related to NK cell differentiation. Similar to NK-like T cells, cytokine-induced killer (CIK) cells represent a heterogeneous population of polyclonal CD56+ T cells that exhibit phenotypic and functional properties of NK cells, and can be expanded from different sources including peripheral blood, bone marrow, and cord blood through expansion protocols involving the timed addition of IFN-γ, anti-CD3 antibodies and IL-2 (110). CIK cells have demonstrated safety and efficacy in several clinical trials where patients with solid cancers were enrolled (111). The combination with chemotherapeutic regimens, anti-immune checkpoint antibodies, or DCs improves the efficacy of CIK cell therapy.

NK-like T cells are categorized as a distinct subset of T cells expressing CD56 that co-express additional NK-related receptors and recognize antigens in a CD1d-independent manner (112, 113). The array of NK-related receptors expressed on NK-like T cells is summarized in **Figure 5B**. They include prototypical activating receptors such as CD16, CD56, and NKG2D, as well as inhibitory NK receptors including CD94, NKG2A, and members of the CD158 killer cell immunoglobulin-like receptor family. Phenotypically and morphologically, NK-like T cells are highly comparable with cytotoxic NK cells with higher expression of perforin and granzyme, except for the expression of TCR-CD3 complex, the low or absent expression of CD16 and the higher expression of the IL-2 receptor- β chain (IL-2R β). 2DG-induced NK-like T cells can exhibit higher cytotoxic activity against solid cancers than conventional T cells *in vitro* and *in vivo* (106, 109). Furthermore, the prominent feature of 2DG-induced NK-like T cells is decreased binding to immunosuppressive tumor-derived galectins by changing N-glycan of CD7 and CD45 on T cells and the decreased binding to PD-L1 by changing N-glycan of PD-1 on T cells (106, 109) (**Figure 5C**). Thus, 2DG-induced NK-like T cells have advantages in cancer therapy through more cytotoxicity and protection from the immunosuppressive TME. In fact, when 2DG-induced NK-like T cells were introduced with CAR against new antigens such as FMR1NB, theses CAR-NK-like T cells effectively killed lung cancer cells *in vitro* and *in vivo* cancer xenograft models of lung cancer (109).

### Overcoming of CAR-T cells limitations in cancer therapy

Limited tumor infiltration and the immunosuppressive TME remain the main challenges that impede the efficacy of CAR-T cell therapy in solid cancers.

### Facilitating CAR-T cells tumor infiltration

Before antigen recognition, CAR-T cells must successfully access tumor sites. CAR-T cells’ migration depends on chemokines secreted by tumor cells and chemokine receptors (CCRs) expressed by effector T cells. Therefore, optimizing CAR-T cell therapy to express appropriate CCRs capable of binding specific chemokines can enhance their infiltration into the TME. In this context, T cells engineered with the chemokine receptor CCR2, binding to its ligand CCL2 (MCP-1), or macrophage colony-stimulating factor-1 receptor (CSF-1R), binding to its ligand CSF1, have an effective trafficking effect to the tumor site (114, 115). In preclinical studies, CCR2 or CSF-1R expressing CAR-T cells significantly enhanced their trafficking to their ligand-producing cancer cells accompanied with cancer cell regression (115, 116). Armed CAR-T cells secreting immunostimulatory cytokines such as IL-12, IL-18, or IL-15, modulate an immunomodulatory microenvironment leading to better CAR-T cell survival and the recruitment of endogenous memory T cells and NK cells (117). Another obstacle that impedes CAR-T cells infiltration to the tumor site is abnormal vascularization. To overcome poor vascularization, targeting the vascular stroma using anti-angiogenic molecules including vascular endothelial growth factor receptor 2 (VEGFR-2), ανβ3 integrin, ανβ6 integrin, is a potential approach (118–121). Notably, integrin anb3 is highly expressed on activated endothelial cells and neo-vessels but not on normal tissues. Inhibition of integrin ανβ3 signaling with antibodies, peptides, peptidomimetics, or other antagonists therefore has great potential in the treatment of cancer (122).

### Overcoming the immunosuppressive tumor microenvironment

Another challenge facing CAR-T cells in solid cancers is the immunosuppressive TME. Immunosuppressive factors in the TME are regulatory T cells (Tregs), myeloid-derived suppressor cells (MDSC), tumor-associated macrophages (TAM), tumor-associated neutrophils (TAN), and tumor-associated fibroblasts, recruited by many cytokines including TGF-b, IL-4, and IL-10 (123) (**Figure 3B**). Therefore, targeting immunosuppressive cells in the TME improves the efficacy of CAR-T cell therapy. Of immunosuppressive cells, Tregs, MDSC, and TAM are the major components of the TME.

Tregs orchestrate immune suppression in the TME through two main mechanisms. First, Tregs secrete inhibitory cytokines including TGF-β, IL-10, and IL-35, which suppress antigen presentation by DCs and inhibit CD4^+^T helper T cell and CD8^+^ T cell function. Second, Tregs secrete perforin and granzyme, which directly kill effector cells such as cytotoxic T cells, NK, and other immune cells (**Figure 3**) (124–126). Tregs in the TME express several cell surface markers including CD25, CTLA-4, PD-1, ICOS, GITR, OX40, CD15s, CCR4 and CCR8, and these markers can be used to deplete Treg cells. Treg depletion enables a self-amplifying loop of T cell activation, which promotes T cell infiltration and cancer destruction in mouse models. However, depletion of Tregs using antibodies or reagents against cell surface markers in the clinic fails to selectively deplete or inhibit Tregs because of the lack of specific targets for tumor-infiltrating Tregs. Further, systemic depletion of Tregs could increase the risk of immune-related adverse events such as autoimmunity-related toxicities. Another strategy for suppressing Treg function in the TME is blocking the migration of Tregs to the TME. Tregs are chemoattracted through chemokine gradients between chemokine receptors including CCR10, CCR8 and CCR4 expressed on Tregs and the corresponding chemokines, including CCL28, CCL1, and CCL22, expressed from target tumor cells in the TME (127). Blocking chemokine and chemokine receptor interactions by antibodies against CCR10, or CCR4, attenuates Treg accumulation into the TME, which could increase anti- cancer immune responses.

MDSCs deplete the essential nutrients for T cells through upregulating arginase 1 which reduces the expression of L-arginine, a substrate necessary for T cell activation and proliferation (128). MDSCs further metabolize L-arginine by iNOS to generate Nitric oxide (NO) and reactive oxygen species (ROS), which suppress T cell activation and induce T cell apoptosis (**Figure 3**) (129). One of the strategies to reduce or eliminate MDSCs in TME is the promotion of further differentiation and maturation of MDSCs into mature myeloid cells such as macrophage or dendritic cells. The differentiation of MDSCs has been achieved with agents such as all-trans retinoic acid, IL-12, RUNX1, and CpG oligonucleotides (128). Alternatively, the inhibition of the intracellular signaling JAK-STAT signaling pathway dampens the suppressive function of MDSCs (130). Recently, Galectin-1 expression was found to be associated with enhanced MDSC phenotypes and poor prognosis in diverse human cancers (131). Galectin-1 secreted from tumor cells and stromal fibroblasts simultaneously orchestrates immunosuppressive and pro-angiogenic programs in MDSCs, because galectin-1 drives tumor growth *in vivo* through glycosylation-dependent interactions with the CD18-CD11b-CD177 receptor complex and STAT3 signaling. Thus, targeting Galectin-1 by Galectin-1-neutralizing antibodies could be a new therapeutic way to overcome the immunosuppressive TME.

TAMs represent one of the tumor-infiltrating immune cell types and are categorized into either of two functionally contrasting subtypes: classical M1 macrophages exerting anti-tumor-function and M2 macrophages promoting the occurrence and metastasis of tumor cells. In the TME, macrophages are polarized towards an M2 phenotype and facilitate tumor progression and metastasis by promoting tumor cell invasion, angiogenesis, and immunosuppression (132, 133). TAMs suppress T cell function through secreting immunosuppressive cues such as IL-10, C-C motif chemokine ligand (CCL)18, CCL24 and transforming growth factor β (TGF-β), that polarize infiltrated immune cells into immunosuppressive phenotype (134). Targeting M2 macrophages is successfully achieved by using folate receptor β (FRβ)-specific CAR-T cells (135). With a growing body of evidence pointing out the relevance of glycosylation in the modulation of macrophages (33), targeting galectins 1 and 3 blocks the M1/M2 macrophage polarization. Blocking of Siglecs-9/10/15 with antibodies enhances the phagocytosis of tumor cells by macrophages (52). Conversely, administration of sialidase to induce desialylation polarizes TAMs to an immune-promoting M1 macrophage phenotype.

### Overcoming CAR-T cells’ toxicities

Cytokine release syndrome (CRS) is a common life-treating inflammatory condition generated by overactivation of CAR-T cell therapy. Cytokines such as IFN-γ, IL-1, IL-6, and IL-10 are associated with CAR-T cells-related CRS (136). Strategies to mitigate CRS include blockade of IL-1 receptor (IL-1R), IL-6, and granulocyte-macrophage colony-stimulating factor (GM-CSF), which have demonstrated some effectiveness in treating CRS. Further studies are required to improve CAR-T cell efficacy and toxicity by extending their persistence, facilitating their trafficking, and improving their infiltration to the tumor site.

Immune effector cell-associated neurotoxicity syndrome (ICANS) is another neurotoxic complication of immune effector cell therapies (137). In CAR-T cell therapy, ICANS typically occurs after the development of CRS and is correlated with the severity of CRS (138). Relying on the abnormal systemic inflammatory response triggered after activation of infused CAR-T cells, ICANS is characterized by increased levels of pro-inflammatory cytokines and marked endothelial activation. However, there remain no approved therapies for the prevention of ICANS.

### Alternative cell source of CAR-T cells in cancer therapy

**Figure 6:**
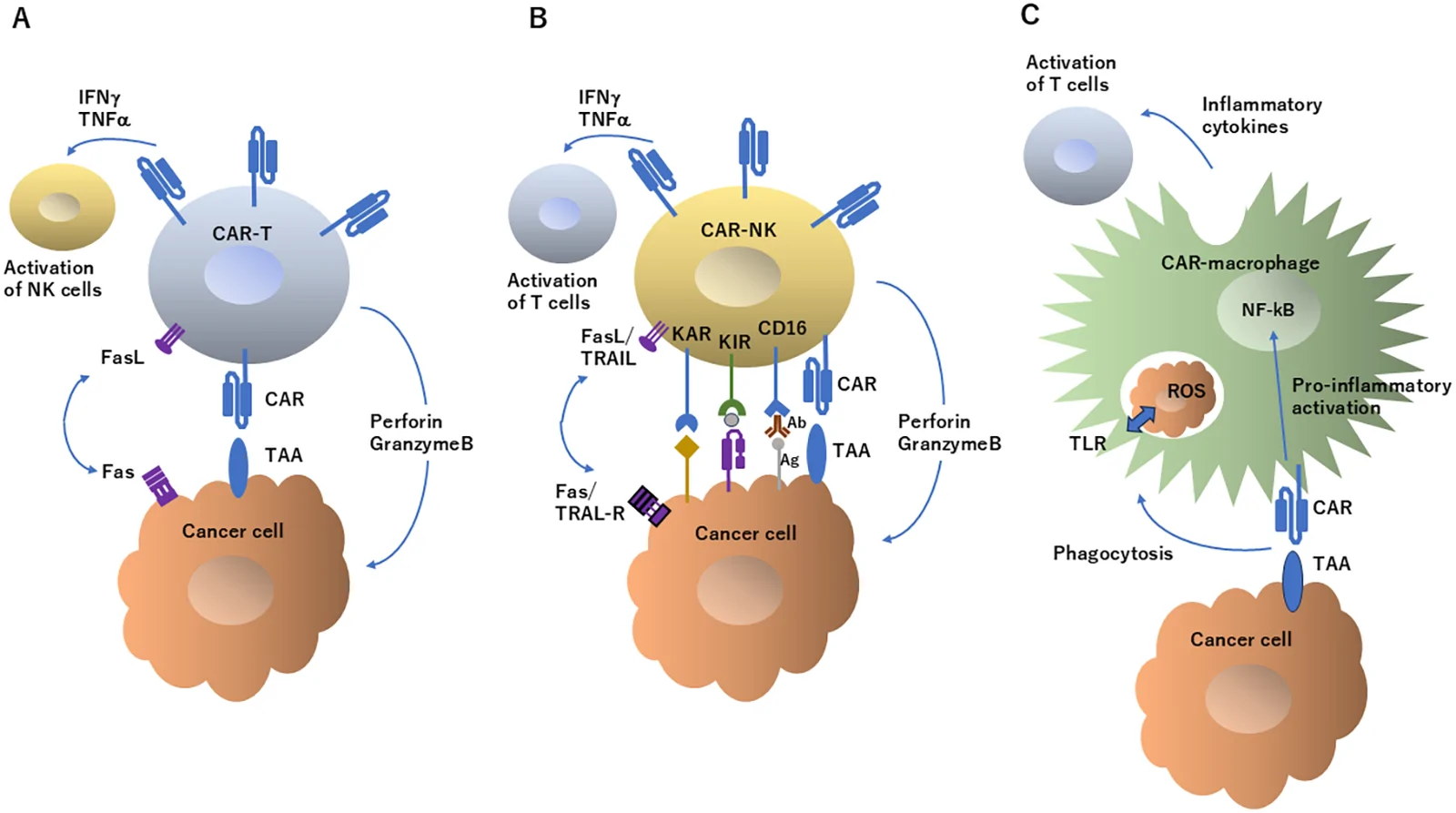
Killing mechanisms of CAR-T, CAR-NK, and CAR-M cells. **(A)** Cancer killing mechanism of CAR-T cells. Activated CAR-T cells specifically recognize the tumor-associated antigen (TAA). Cytotoxicity of CAR-T cells is mediated by perforin and granzyme B granules secretion and by death receptor pathway Fas/Fas-L leading to apoptosis of cancer cells. Activated CAR-T cells also secrete IFN-γ and TNF- α which promote Natural Killer (NK) cell anti-tumor cytotoxic activity. **(B)** Cancer killing mechanism of CAR-NK cells. The activity of CAR-NK cells is regulated by the killer activating (KAR) and killer inhibitory receptors (KIR) on NK cells. Cytotoxicity of CAR-NK cells is mediated by perforin and granzyme B granules secretion and by death receptor pathway Fas/Fas-L and TRAIL/TRAL-R leading to apoptosis of tumor cells. CAR-NK cells trigger ADCC through the CD16 Fc receptor which recognizes antibody-opsonized tumor cells. Activated CAR-NK cells also secrete IFN-γ and TNF-α which promote T cell anti- cancer cytotoxic activity. TRAIL, TNF-related apoptosis-inducing ligand **(C)** Cancer killing mechanism of CAR-M. The binding of a specific TAA with CAR receptor on CAR-M generates activation signals mediating tumor phagocytosis, activation of transcription factors such as NF-kB and subsequent release of pro-inflammatory cytokines, which activate T cell-mediated immunity against tumor. TLR, Toll-like receptor; ROS, reactive oxygen species.

The efficacy of CAR-T cell therapy in solid cancers is further limited by heterogeneous cancer antigen expression, lack of tumor-specific targets, poor tumor infiltration, and the immunosuppressive TME. Consequently, increasing interest has emerged in engineering CAR constructs in alternative immune cell types. Natural killer (NK) cells and macrophages (M) are potential candidates for immune cell therapy (55, 139, 140) (**Figure 6**).

### CAR-NK cells in cancer therapy

With respect to CAR design, most constructs used for NK-cell engineering share a similar extracellular structure with those used in CAR-T cells but incorporate NK-specific intracellular signaling domains, such as NKG2D and DNAX-activation protein (DAP) 10 or DAP 12, to improve cytotoxicity. NK cells possess several advantageous characteristics, including non-MHC-restricted recognition, an inherent ability to infiltrate tumor tissues, potent cytolytic ability, and lower risk of adverse effects, especially CRS, graft-versus-host disease (GvHD), and ICANS. Although NK cells use cytotoxic mechanisms similar to those of T cells to kill tumor cells, their target-recognition mechanisms differ (141) (**Figure 6B**). NK cells recognize malignant cells via multiple signals arising from different cell surface receptors, including killer activating and killer inhibitory cell immunoglobulin-like receptors (KARs and KIRs) (142). The recognition mechanisms to target cells are inhibitory receptors of NK cell surface, which interact with class I molecules of the MHC. The attack of NK cells on healthy cells is actively suppressed by a recognition of self-MHC molecules that delivers an inhibitory signal, while NK cells kill cells that lose their MHC class I molecules. Thus, while CAR-T cells only kill cells that have specific target antigens, CAR-NK cells exhibit intrinsic cytolytic activity, thus they kill even cancer cells that do not express the target antigens. Moreover, NK cells are key mediators of ADCC recognizing the IgG Fc fraction bound to tumor cells and kill cancer cells through Fc receptors FcgRIII (CD16). Unlike CAR-T cells, CAR-NK cells do not cause severe toxicities such as CRS and ICANS (143). This is partly owing to a differential cytokine secretion profile; activated NK cells produce IFN-γ and GM-CSF, whereas activated T cells predominantly produce IL-1, IL-6, TNF-α or Monocyte Chemoattractant Protein-1 (MCP-1), all of which are associated with CRS and severe neurotoxicity (144). Consequently, CAR-NK cells could be safer for clinical applications, compared with CAR-T cells. However, NK cells have a short half-life, which means that CAR-NK is advantageous in the event of severe toxicity, but also poses a challenge in that repeated dosing may be required to achieve a durable response. Siglec-7 and Siglec-9 are abundant in NK cells and inhibit NK cytotoxicity by interacting with sialoglycans in the TME (48). Administration of sialidase to induce desialylation significantly enhances NK cell-mediated cytotoxicity and inhibits tumor progression in leukemia and cervical cancer (145). Thus, targeting the sialoglycan-Siglec-9 pathway enhances anti-tumor immunity *in vitro* and *in vivo* (146).

### CAR-M cells in cancer therapy

CAR-M cells have recently emerged as competitive candidates for the treatment of solid tumors owing to their intrinsic phagocytic properties, antigen-presentation ability, and natural propensity to infiltrate the ECM (147, 148) (**Figure 6C**). Tumor-associated macrophages undergo activation into M1 or M2 phenotypes (149). Peripheral blood mononuclear cell-derived M1 macrophages are characterized by the production of pro-inflammatory factors such as IL-8, IL-6, and TNF-a, as well as elevated expression of inflammatory surface markers including natriuretic peptide receptor (NPR), CD14, and CD68. In response to solid tumors, macrophages can differentiate into M1 macrophages that exert antitumor activity by phagocytosing tumor cells and releasing reactive oxygen and nitrogen species following Toll-Like Receptors (TLR) activation. M1 macrophages also release IL-12, which initiates NK-cell cytotoxicity and stimulates Th1 and tumor-specific CD8+ cytotoxic T cell response (150). Thus, converting M2 into M1 macrophages is a promising immunotherapeutic approach for solid tumors. Macrophages are able to remodel the ECM via secreting matrix metalloproteinases. Thus, CAR-M cells, once infiltrated into the TME, can exert their cytotoxic function against tumor cells through a CAR-dependent and independent pathway. Similar to CAR-NK cells, CAR-M cells have a low risk of GVHD owing to rapid extravasation from blood vessels and *in vivo* limited expansion capacity. However, the application of CAR-M cells in the clinic remains limited, because of the potential to induce CRS and off target toxicity (151). Therefore, further investigations are needed to optimize CAR-M cell production and ensure their safety.

### Potential combination therapy to enhance CAR-T cell therapy

Owing to the limited efficacy of CAR-T cell therapy for solid tumors, combinations with chemotherapy, radiotherapy as well as immune checkpoint inhibitor therapy have been tested to synergize CAR-T cell therapy (152). Chemotherapy at low doses increases tumor antigens on tumor cells, which allows CAR-T cells to interact with tumor cells. Chemotherapy also down-regulates Treg and MDSCs, leading to promotion of CAR-T cell proliferation, and their persistence in the TME (123). Radiotherapy up-regulates tumor antigens on tumor cells allowing maturation and activation of dendritic cells, which associate with more presentation of tumor antigens to T and CAR-T cells. Radiotherapy also induces chemokines, IFN-γ, and damage-associated molecular patterns (DAMPs) release by tumor cells, leading to increased migration and infiltration of CAR-T cells (153). Oncolytic viruses promote tumor debulking which enhances CAR-T cell infiltration, proliferation and activation, and induces proinflammatory cytokinin production (154). Thus, viruses increase tumor death through a direct effect and enhanced CAR-T cell activity. Immune checkpoint inhibitors reduce CAR-T cells exhaustion through repressive pathways, particularly in tumors with low immunogenicity. However, clinical efficacy remains variable (155).

Exploiting the antigen cross presentation ability of dendritic cells to potentiate host effector and memory CD8 T cells responses is crucial for an adaptive immune response against tumors. Generally, exogenous antigens are internalized and are degraded within endosome/lysosome, which engenders antigen presentation onto MHC class I and induction of humoral immunity (156). The antigen cross presentation capacity of dendritic cells in tumor-bearing hosts is often defective, which causes an ineffective tumor-specific cytotoxic T cell response. One strategy to induce cross presentation is cytosolic delivery of an exogenous antigen using fusogenic or endosomolytic molecule-induced nanocarriers, especially pH-responsive polysaccharides (157). Antigen-loaded liposomes exhibit strong adjuvant effects and induce the increase of dendritic cells, M1 macrophages and effector T cells in the TME. Thus, administration of antigen-loaded liposomes induces antigen-specific cellular immunity, leading to potentiate CAR-T cell therapy.

## Concluding remarks

Immunotherapy, which aims to boost the host immune system to eliminate malignant cells, represents a landmark breakthrough in cancer treatment. Immunotherapies, including immune checkpoint inhibitors and adoptive cell transfer, have achieved inspiring results. However, all these treatments in the clinical practice have their own limitations. Accumulating evidence has revealed that the translational modification, especially glycosylation, is one of the critical regulators in immunotherapies. In fact, PD-1 and PD-L1 are heavily N-glycosylated, which stabilizes PD-1/PD-L1 interaction. Targeting the N-glycosylation significantly increases the efficacy of PD-1/PD-L1 blockade, thereby enhancing immune checkpoint inhibitors therapy.

Despite the remarkable success of CAR-T cell therapy in hematological malignancies, its therapeutic efficacy in solid cancers remains poor, because they lack cancer -associated antigens, have an immunosuppressive TME, and other problems lead to solid tumors not responding to CAR-T cell therapy. To overcome obstacles in CAR-T cell therapies for solid cancers, one of the strategies is the optimization of CAR-T cell cultures through intervening during CAR-T cell expansion stages. These optimized culture conditions are related to the regulation of T cell metabolism through the increase in OXPHOS and appropriate inhibition of glycolysis *in vitro*. These processes lead to a high percentage of stem cellular memory T cells and central memory T cells, ultimately resulting in superior longevity and anti- cancer potential for CAR-T cell immunotherapy. Several metabolites, such as glutamine inhibitors and 2-DG which enhance OXPHOS and restrict glycolysis, and other drugs which regulate intracellular signaling, have been applied for clinical trials to promote T cell differentiation and prolong persistence. Along these lines, our laboratory demonstrated the immune-active effect of 2DG through the inhibition of N-glycosylation. T or CAR-T cells treatment with 2DG leads to acquisition of NK-like T cell properties; showing more cytotoxicity with higher expression of perforin and granzyme, and more protection from tumor-cell toxicity by decreasing binding to immunosuppressive tumor-derived galectins.

The differentiation and proliferation of immune cells are deeply dependent on glycosylation, in that glycosylation of surface molecules is required for the interaction between cells and binding to their ligands. Cytokines such as IL-2 or IL-7 regulate the glycosylation profiles on immune cells through changing the various glycosyltransferase activities. Further, potent mediators, including glycan-binding proteins such as galectin, lectins, or Siglecs, affect immune cell activities through binding to glycan-coated surface molecules. Thus, the modification of glycosylation would be an alternative way to improve the immune-activities of CAR-T cells against solid cancers.

Besides the role of glycosylation in the immune cell biology, abnormal glycosylation is a widely recognized hallmark of cancer and impacts the anti-tumor response of immune cells in the TME. Therefore, abnormal glycosylation has emerged as a promising target for immunotherapy, and targeting abnormal glycosylation can reshape the TME and promote the immune response. A significant number of glycoproteins unique to solid tumors have been identified as TACAs, which are excellent antigenic targets for CAR-T cells. Further, the glycan-binding antibodies and the non-antibody glycan binders such as lectin present exciting new approaches, and glycan-binding CAR-T cells have shown preliminary successes in targeting and treating solid malignancies without significant adverse effects.

Another challenge facing immunotherapy in a solid tumor is an immunosuppressive TME. Immunosuppressive factors are derived from Tregs, MDSCs, and TAMs accumulating in the TME. The infiltration, differentiation, and proliferation of these immunosuppressive cells are dependent on the different glycosylation profiles, including N-glycan, O-glycan, sialylation, and fucosylation. Therefore, it is possible that new agents capable of modifying glycosylation profiles in the TME could exclude immunosuppressive cells from the TME, thereby overcoming its immunosuppressive effects.

In conclusion, as the regulation of glycosylation in cancer immunity has received extensive attention, an in-depth study of the molecular mechanism of glycosylation-mediated immune escape and discovery of new effective drug targets are expected to promote the development of next-generation cancer immunotherapy.
